# Viral FLICE inhibitory protein of Rhesus monkey rhadinovirus inhibits apoptosis by enhancing autophagosome formation

**DOI:** 10.1186/1750-9378-7-S1-P44

**Published:** 2012-04-19

**Authors:** Krit Ritthipichai, Yuchen Nan, Ioannis Bossis, Yan-Jin Zhang

**Affiliations:** 1 Molecular Virology Laboratory, University of Maryland, College Park, MD, USA; 2 Cell Biology Laboratory, VA-MD Regional College of Veterinary Medicine and Maryland Pathogen Research Institute, University of Maryland, College Park, MD, USA

## 

Rhesus monkey rhadinovirus (RRV) is a gamma-2 herpesvirus closely related to human herpesvirus 8 (HHV8). RRV encodes viral FLICE inhibitory protein (vFLIP), which has death effector domains. Little is known about RRV vFLIP. This study intended to examine its function in apoptosis. Here we found that RRV vFLIP inhibits apoptosis induced by tumor necrosis factor-α (TNF-α) and cycloheximide. In HeLa cells with vFLIP expression, the cleavage of poly [ADP-ribose] polymerase 1 (PARP-1) and activities of caspase 3, 7, and 9 were much lower than those in controls. Cell viability of HeLa cells with vFLIP expression was significantly higher than control cells after apoptosis induction. However, RRV vFLIP appears unable to induce NF-κB signaling when tested using NF-κB reporter assay. RRV vFLIP was able to enhance cell survival under starved conditions or apoptosis induction. At early time points after apoptosis induction, autophagosome formation was enhanced and LC3-II level was elevated in cells with vFLIP and, when autophagy was blocked with chemical inhibitors, these cells underwent apoptosis. Full length of vFLIP is needed for the function against apoptosis as truncation variants of vFLIP were unable to block apoptosis induction. Moreover, RRV latent infection of BJAB B-lymphoblastoid cells protects the cells against apoptosis by enhancing autophagy to maintain cell survival. Knockdown of vFLIP expression in the RRV-infected BJAB cells with siRNA abolished the protection against apoptosis. These findings indicate that vFLIP protects cells against apoptosis by enhancing autophagosome formation to extend cell survival. The finding of vFLIP’s inhibition of apoptosis via the autophagy pathway provides insights of vFLIP in RRV pathogenesis.

